# Shifts in Microbial Community Structure and Co-occurrence Network along a Wide Soil Salinity Gradient

**DOI:** 10.3390/microorganisms12071268

**Published:** 2024-06-22

**Authors:** Yan Li, Juan Wang, Eryang Li, Xiaodong Yang, Jianjun Yang

**Affiliations:** 1 College of Ecology and Environment, Xinjiang University, Urumqi 830017, China; liyan1006@xju.edu.cn (Y.L.);; 2 Key Laboratory of Oasis Ecology, Ministry of Education, Urumqi 830017, China; 3 Chengdu Institute of Biology, Chinese Academy Sciences, Chengdu 610042, China; 4 Department of Geography & Spatial Information Technology, Ningbo University, Ningbo 315211, China

**Keywords:** salinization, community composition, key species, soil remediation

## Abstract

The response of microbiomes to salinity has been clarified in different geographic scales or ecosystems. However, how soil microbial community structure and interaction respond to salinity across wide salinity range and climatic region is still unclearly resolved. To address this issue, we examined the microbial community’s composition in saline soils from two climatic regions (coastal wetland and arid desert). Our research confirms that soil salinity had a negative effect on soil nutrient content. Salinity decreased the relative abundance of bacteria, but increased archaea abundance, leading to the shifts from bacteria dominant community to archaea dominant community. Low-water medium-salinity soil (LWMS) had the most complex archaeal community network, whereas for bacteria, the most complex bacterial community network was observed in low-water high-salinity soils (LWHS). Key microbial taxa differed in three salinity gradients. Salinity, soil water content, pH, total nitrogen (TN), and soil organic carbon (SOC) were the main driving factors for the composition of archaeal and bacterial community. Salinity directly affected archaeal community, but indirectly influenced bacteria community through SOC; pH affected archaeal community indirectly through TN, but directly affected bacterial community. Our study suggests that soil salinity dramatically influences diversity, composition, and interactions within the microbial community.

## 1. Introduction

Soil salinization is a widespread issue. More than 900 million hectares of soil worldwide have been identified as being at risk of salinization driven by natural or anthropogenic processes [1]. Furthermore, the area of saline-alkaline land is increasing by approximately 1–1.5 million hectares annually [2]. Soil salinization is a detrimental process that causes the loss of topsoil and nutrient depletion due to the reduction in soil structure and decreased vegetation cover [3], low soil fertility, poor structure, and an imbalance between input and output [4]. Microbiomes play a crucial role in maintaining soil ecosystem quality, function and health [5,6]. They improve soil structure and aggregation by producing substances such as glues and polysaccharides that bind soil particles together. They also play a key role in nutrient cycling processes such as nitrogen fixation, nutrient mineralization, organic matter decomposition, and soil fertility [7].

Plenty of researches have demonstrated the effects of soil salinization on soil microbial community at different geographic scales, salinization stages, and in various ecosystems, including farmland [8,9], salt lake sediments [10,11,12], wetlands [13,14,15], and deserts [16,17,18], these evidences lead to the conclusion that soil salinization leads to a decrease in microbial diversity and alterations to the structure of microbial community [19], which is probably due to a filtering effect that favors salt-tolerant or halophilic microorganisms over salt-sensitive taxa [20]. However, many of the studies were conducted on specific ecosystems or in limited salinity ranges. Broad-scale investigation of microbial community composition, function and driving factors across different climatic, geographic and salinity ranges is still inadequate. Yang et al. carried out a survey in surface sediments of the Qinghai-Tibetan Lakes which provided insights into the composition of bacteria and archaea changes with salinity from saline to hypersaline (salinity from 0.6 to 324 g·L^−1^) [10]. Liang et al. investigated the biogeographical patterns of bacterial communities in saline soils of northeast China at a large salinity range (8.9 mS/cm to 352 mS/cm), but this research focused on bacteria, rather than Archaea [20]. Archaea are a distinct group of organisms with unique genetic, biochemical and physiological characteristics that thrive in extreme environments [21]. They exhibit a wide range of metabolic diversity, including methanogenesis [22] and biogeochemical cycles, such as nitrogen cycling [23,24]. Therefore, a comprehensive investigation encompassing a wide range of salinity and a broad-geographic scale, which includes both bacteria and archaea, is essential to deepen our understanding of the impact of salinity on microbiomes composition, function, and ecosystem management.

In the soil community, most microorganisms may interact with each other to establish diverse relationships, including mutualism, commensalism, synergism, competition, parasitism, and predation. These relationships shape the structure and function of microbial communities [25]. Co-occurrence network analysis provides a powerful tool for exploring the complexity of microbial communities, revealing hidden relationships which are more difficult to analyze using traditional analytical approaches, and helping identify keystone taxa that play crucial roles in maintaining community structure and function. These taxa may have a disproportionate impact on the overall ecosystem dynamics and could be important targets for conservation or manipulation in microbial communities [25,26,27]. Clarification of the microbial community’s network characteristics and its response to salinity are important to advance our understanding of the ecological principles governing microbial interactions and screen keystone species.

China is the third-largest country in the world in terms of saline-alkali land [16,28]. In China, saline-alkali soils are distributed across the entire country [29] which is divided into eight zones according to the natural geographical conditions and soil formation processes: the coastal humid to semi-humid tidal saline-alkali zone, the northeast semi-humid to semi-arid grassland-meadow saline-alkali zone, the Huang-Huai-Hai semi-humid to semi-arid dry farming grassland saline-alkali zone, the Gansu-Xinjiang desert saline-alkali zone, the Qinghai extreme desert saline-alkali zone, and the Tibet high cold desert saline-alkali zone [30]. The arid and semi-arid regions in northern China and the coastal region represent the two main distribution areas [29]. Due to unique geographical environments and arid or semi-arid climate in northern China, soil salinization is increasingly worsening [28]. Whereas the coastal areas in eastern to southeastern China have humid to semi-humid climate, the formation of large areas of natural saline-alkali land is mainly driven by seawater immersion and soil leaching. Climate, terrain and landforms, parent material, hydrological conditions, and biological (vegetation) conditions drive the soil saline-alkaline process that subsequently has a profound impact on microbial community diversity, composition, and microbe-mediated nutrient cycle and soil quality [29]. Previous studies have revealed the microbial community in coastal estuarine wetland ecosystems and arid lands or high-altitude plateaus, providing insights into microbial communities’ composition or functions under specific salinity conditions or gradients [10,13,14,16,31]. However, there is still a paucity of knowledge regarding the ensemble understanding of how the microbial community’s structure and microbiomes’ interaction respond to a wide salinity range, the extent of the differences in microbial communities between distinct climatic conditions (i.e., precipitation), and the effects of soil variables on microbial community assembly.

Therefore, in the present study, based on our previous studies in arid saline soil, we further collected natural saline soils from coastal regions in China and examined the microbial community’s composition by employing a high-throughput sequencing technique to gain insights into the above-mentioned questions. The aims of this study were to reveal the microbial community’s shifts in a wide range of salinity, the change in community structure driven by distinct climatic induced salinization process, and the effects of soil salinization on microbes’ interactions, based on which, the study discussed the potential values for soil restoration.

## 2. Materials and Methods

### 2.1. Studying Area

The soils were collected from the saline drylands (Xinjiang, northwest China) and coastal wetlands (Shandong, Jiangsu, Zhejiang, and Fujian provinces in southeast China) where the climate and precipitation conditions are distinct. Xinjiang is located deep inland with a distinct temperate continental climate, having a dry and arid climate with low precipitation (with an average of around 150 mm). Southeast China is mostly located in the subtropical monsoon climate zone with high rainfall (annual precipitation of about 750–1700 mm). This promotes groundwater replenishment of the soil water, creating the driving conditions for the upward movement of soil salinity. At the same time, soil leaching is enhanced, and the land gradually becomes saline-alkali soil due to seawater immersion and salt leaching by rainwater [32].

### 2.2. Soil Sampling

**Figure 1:**
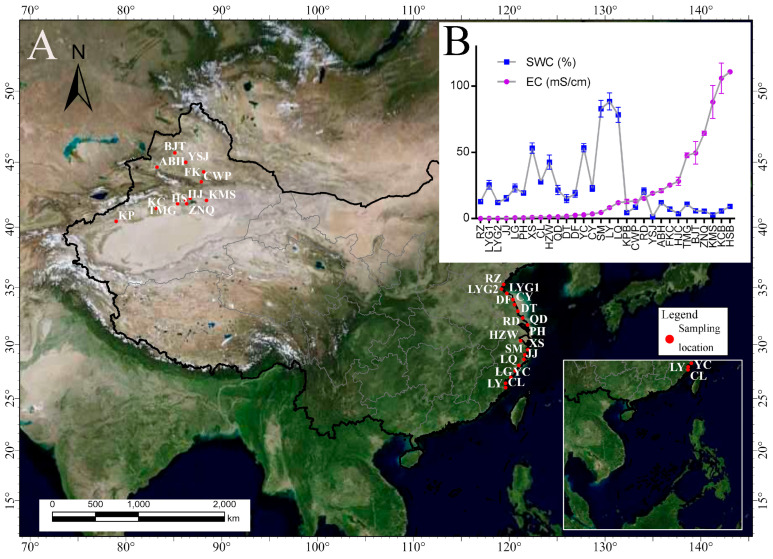
Geographic location of soil samples used in present study. (**A**), locations of sampling site; (**B**), the curves of soil water content (SWC) and electronic conductivity (EC) across all samples.

A total of 90 samples were collected from 30 locations in September 2021 from Xinjiang, and in August 2022 from coastal area (Figure 1, Table S1). As the sampling sites cover southeast and northwest China where the climate is distinct. A relative consistent temperature and precipitation condition (no rainfall ahead of sampling day) was maintained between the two sampling months to eliminate the community variation caused by difference of climate factors as far as possible. A 10 × 10 m quadrat was set at each site, from which three samples were collected at 0–20 cm layer and stored in a 50 mL sterile tube after excluding any stones or plant debris. The soil samples for metagenome analysis were kept at approximately −10 °C in a vehicle fridge during the collection process that was completed within ~4 days in the Xinjiang and the southeast China, respectively. When samples were transported to the laboratory, they were divided into two parts, one part was used for physicochemical variable determination after being air-dried and sieved with a 2 mm mesh, while the other was subjected to genomic DNA isolation as soon as possible. If DNA extraction could not be completed in a timely manner, soil samples were temporarily stored at −80 °C for a few days. 

### 2.3. Soil Properties Determination

The soil properties were determined according to Yang et al. [16]. Briefly, the soil water content (SWC) was determined by gravimetric method after soils drying at 105 °C for consistent weight. The pH was measured using an electrode pH meter (DDSJ-319L, Shanghai, China) in a 1:2.5 soil suspension (*w*/*v*). The soil organic carbon (SOC) was measured by potassium dichromate (K_2_Cr_2_O_4_) oxidation method and colorimetric analysis was performed using a UV-1200 spectrophotometer (AOE Instruments, Shanghai, China). The total phosphorus (TP) and available phosphorus (AP) were measured by the Mo-Sb colorimetric method after soil digested with a perchloric acid and concentrated sulfuric acid (HClO_4_-H_2_SO_4_) solution for 60 min. The total nitrogen (TN) was analyzed by an AA3 flow analyzer (SEAL Analytical GmbH, Norderstedt, Germany) after digestion with H_2_SO_4_ and HClO_4_. The nitrate–nitrogen (${NO}_{3}^{-}$-N) and ammonium–nitrogen (${NH}_{4}^{+}$-N) were measured by the AA3 flow analyzer after extraction in 1 mol/L KCl.

### 2.4. Metagenomic Sequencing, Assembly, and Annotation

Genomic DNA was extracted from ~0.5 g soil using a FastDNA SPIN Kit for Soil (MP Biomedicals, Cleveland, OH, USA) following the kit instructions. The quantity and quality of extracted DNA were determined by using a NanoDrop 2000 spectrophotometer (Thermo Fisher Scientific, Waltham, MA, USA) and 1% agarose gel electrophoresis, respectively. The qualified DNA was used for library construction and metagenomic sequencing consulting Yang et al. [16]. In brief, the genomic DNA was randomly sheared into short fragments of about 350 bp. The obtained fragments were end-repaired, A-tailed and ligated with an Illumina adapter. The fragments with adapters were PCR amplified, size selected, and purified. The paired-end library was prepared using an Ultra DNA Library Prep Kit for Illumina (New England Biolabs, Ipswich, MA, USA) and checked with a Nanodrop 2000 and quantified using real-time PCR. The quantified libraries were pooled and sequenced using an Illumina HiSeq platform at Novogene (Tianjin, China).

The raw reads were filtered with Readfq (V8, https://github.com/cjfields/readfq, accessed on 20 August 2023) to obtain clean data that were used for assembly performed in MEGAHIT software (v1.0.4-beta) [33,34], with the following parameter settings: -presets meta-large (-end-to-end, -sensitive, -I 200, -X 400). Scaftigs without N were acquired by breaking the scaffolds from the N junction. MetaGeneMark (V2.10, http://topaz.gatech.edu/GeneMark/, accessed on 20 August 2023) was used to perform ORF prediction for Scaftigs (≥500 bp) of each sample, and the predictions with a length less than 100 nt were filtered out. Redundancy elimination was implemented by the CD-HIT [35] to obtain non-redundant initial gene catalogue with parameter settings: -c 0.95, -G 0, -aS 0.9, -g 1, -d 0. Then, the clean reads were aligned to the initial gene catalogue by using the Bowtie2 to calculate the number of reads of the genes on each sample alignment, with parameter settings: -end-to-end, -sensitive, -I 200, -x 400. Genes with reads ≤2 in each sample were filtered out to finalize the gene catalogue (unigenes) for subsequent analysis [36,37]. The gene abundance table in each sample was calculated based on the number of reads aligned and the length of gene. The blastp of unigenes with those of bacteria, fungi, archaea, and viruses extracted from NCBI’s NR database was implemented by DIAMOND software (V0.9.9) with a cutoff e-value of 10^−5^ [38]. The MEGAN4 software was adopted to determine the species annotation information of unigenes [39]. With the gene abundance table and annotation information, we acquired the abundance at each taxonomy in each sample. The functional annotation of unigenes was performed with DIAMOND software (V0.9.9) against the databases including the KEGG database and eggNOG [40,41]. The best Blast Hit results were selected for subsequent analysis.

### 2.5. Statistical Analysis

The diversity indices were calculated using the vegan package (V2.15.3). One-way ANOVA and Kruskal–Wallis tests were conducted to test for differences in soil variables, microbial community alpha diversity and taxa abundance between salinity gradients. The compositional differences in microbial communities based on Bray–Curtis distances were calculated and visualized through Principal Coordinate Analysis (PCoA) with the ape package (V5.0) [42]. PERMANOVA analysis with the Bray–Curtis similarity matrix was employed to test the similarities between salinity groups. Linear Discriminant Analysis (LDA) effect size (LEfSe) was performed to identify the microbial taxa contributing to difference among the three salinity gradients. Redundancy Analysis (RDA) and mantel analysis were used to quantify the relationship between the microbial community and soil parameters. A co-occurrence network was constructed based on strong significant correlations (absolute value of Spearman’s ρ > 0.7, *p* < 0.01) to explore the interactions between genera with a relative abundance ≥ 0.1%. In addition, Hmisc V4.2-0 was used to calculate the correlation matrix (Spearman correlation coefficient values). The co-occurrence network was visualized in Gephi (V0.92, https://gephi.org/). The keystone taxa (Network hubs, module hubs, and connectors) in the network were identified with z-score and c-score [43]. However, based on these criteria, we only found keystone bacteria in LWMS, and keystone archaea taxa in LWHS soil. As the betweenness centrality (the ability of a node to control the shortest path to other nodes) can be used to identify key species in the network [44]. Therefore, we additionally selected nodes with high betweenness centrality (top 10%) as keystone species. Finally, we employed Structural Equation Model (SEM) with the support of stepwise regressions analyses to test for the direct and indirect effects of influencing factors on soil microbial community structure which was implemented in the package piecewiseSEM V2.3 [45].

## 3. Results

### 3.1. Changes in Soil Variables in Three Salinity Gradients

**Figure 2:**
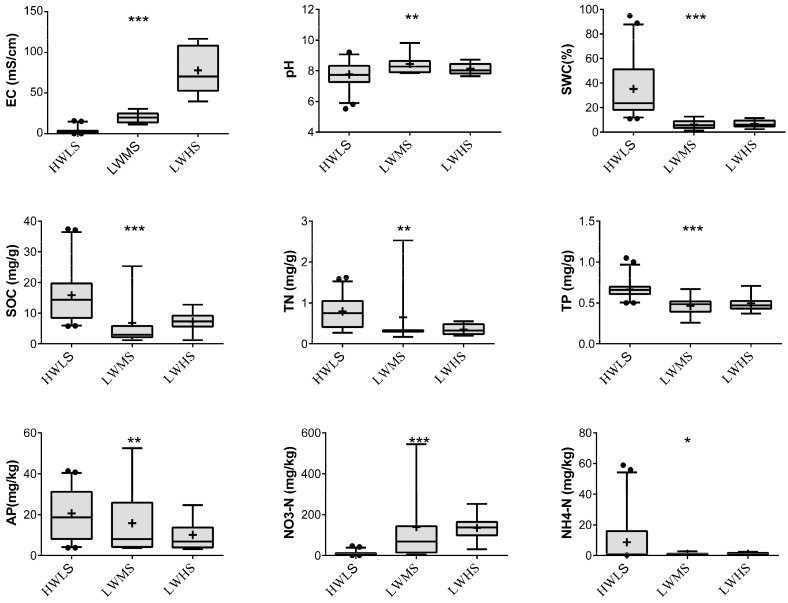
Statistics of soil variables in the three salinity gradients. The dots in each barplot represent outliers, and plus sign indicate mean value of soil variable. *, **, and *** indicating significant differentiation among the three salinity gradients at *p* < 0.05, *p* < 0.01, and *p* < 0.001, respectively.

Soil salinity (EC) differed significantly in the three soil groups (*p* < 0.05), meanwhile, other soil chemical properties varied significantly among the three salt gradients (Figure 2). With the increase in salinity, the content of soil water content (SWC), soil organic carbon (SOC), total nitrogen (TN), total phosphorus (TP), and available phosphorus (AP) decreased (*p* < 0.05); In contrast, the nitrate nitrogen (${NO}_{3}^{-}$-N) was negatively correlated with EC (*p* < 0.05). However, soil pH and ammonium nitrogen (${NH}_{4}^{+}$-N) showed a non-significant positive or negative relationship with EC (*p* > 0.05). These indicated that soil salinization inhibited soil nutrient quantity and had negative effects on soil quality.

### 3.2. Sequencing Data and Metagenome Assembly

This study newly generated 540.55 Gbp of raw data by metagenomic sequencing from 54 coastal wetland soil samples, with an average of 10.01 Gbp per sample. After quality control, a total of 540.16 Gbp clean reads was retained with an average of 10.0 Gbp of each sample. A total of 73.89% of the total genes were annotated on NR, and 14.15% were annotated as unclassified. A total of 57.54%, 48.57%, 42.61%, 37.12%, 34.52%, 31.43%, and 25.72% were annotated on the kingdom, phylum, class, order, family, genus, and species level, respectively. Additionally, we included previously sequencing data (595.64 Gbp of raw reads, 36 samples, 16.31 Gbp per sample) with saline soils in arid regions [16]. Therefore, a total of 1135.8 Gbp of clean data were used for subsequent analysis in the present study. The detailed information of sequencing data used in this research are listed in Table S2.

### 3.3. Microbial Community Composition and Structure

**Figure 3:**
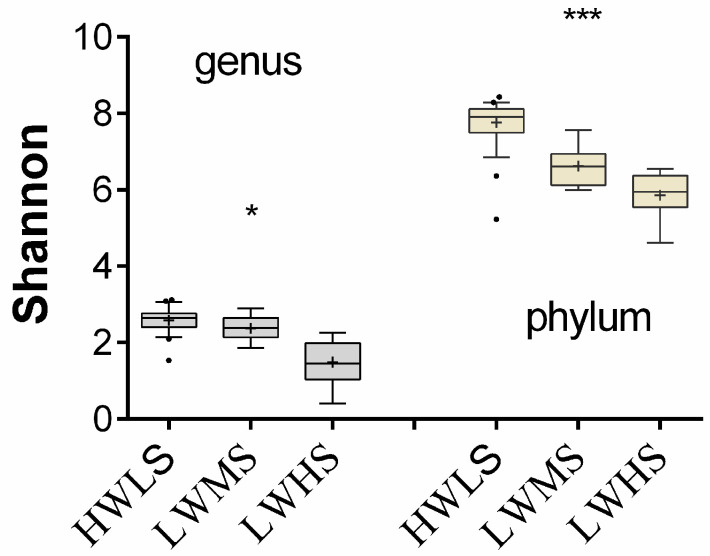
Shannon index in three saline gradients. The dots in each barplot represent outliers, and plus sign indicate mean value shannon index. *, and *** indicating significant difference at *p* < 0.05, and *p* < 0.001.

The Shannon index ranged from 0.404 to 3.118 with a mean of 2.325 ± 0.56 at the genus level, and from 4.616 to 8.426 with an average of 6.144 ± 0.95 at the phylum level (Figure 3). There was a significant difference in Shannon index among the three salt gradients at genus level (*p* < 0.05) and phylum level (*p* < 0.01). The Shannon index was 2.585 ± 0.287, 2.378 ± 0.305, and 1.491 ± 0.566 in HWLS, LWMS, and LWHS, respectively (Figure 3). These findings indicated soil salinization reduced microbial community diversity.

On average, 69.06% of genes were affiliated with bacteria, 16.21% were identified as archaea, 0.054% belonged to Eukaryota, and viruses accounted for 0.21%. At the phylum level, the unigenes were mainly assigned into Euryarchaeota (accounting for 15.16% of the total unigenes), Actinobacteria (11.77%), Proteobacteria (28.38%), Bacteroidetes (4.68%), Acidobacteria (3.13%), Chloroflexi (4.78%), Firmicutes (1.22%), Gemmatimonadetes (2.74%). At the genus level, the most abundant genera were *Nitriliruptor* (1.88%), *Halalkalicoccus* (1.40%), *Gemmatimonas* (1.36%), *Natronomonas* (1.13%), *Woeseia* (1.06%), *Gillisia* (0.78%), *Nocardioides* (0.61%), *Haladaptatus* (0.52%), *Streptomyces* (0.49), *Halomicrobium* (0.46%), *Halorientalis* (0.40%), *Halococcus* (0.38%), *Pseudomonas* (0.38%), *Halopiger* (0.37%), and *Desulfuromonas* (0.37%).

### 3.4. Microbial Community Structure Difference at Salinity Gradients

**Figure 4:**
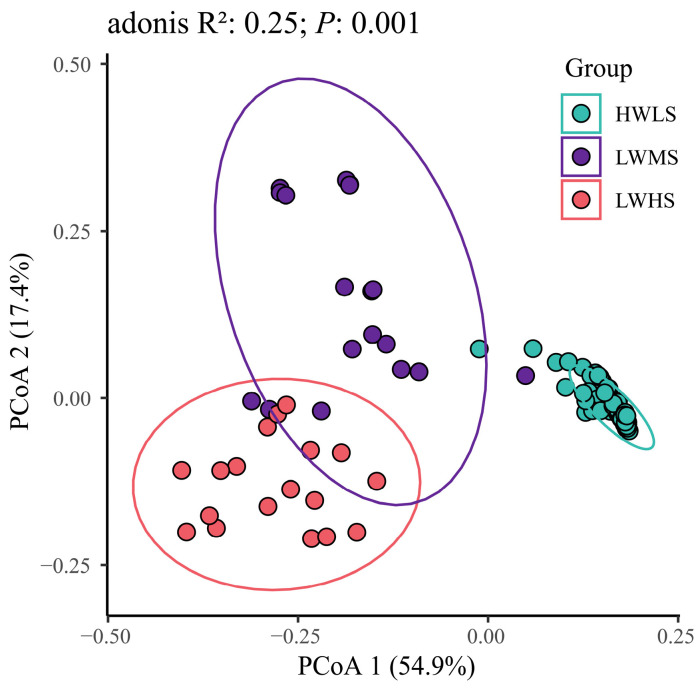
PCoA based on the Bray–Curtis distance matrices indicating β-diversity differences between three salinity groups at genus level.

The microbial communities structure showed a significant difference between the three gradients, in that they were clearly separated along the two axes in the PCoA plot (PERMANOVA R^2^ = 0.25, *p* < 0.001 at genus level and R^2^ = 0.3, *p* < 0.001 at phylum level) (Figure 4, Figure S1).

**Figure 5:**
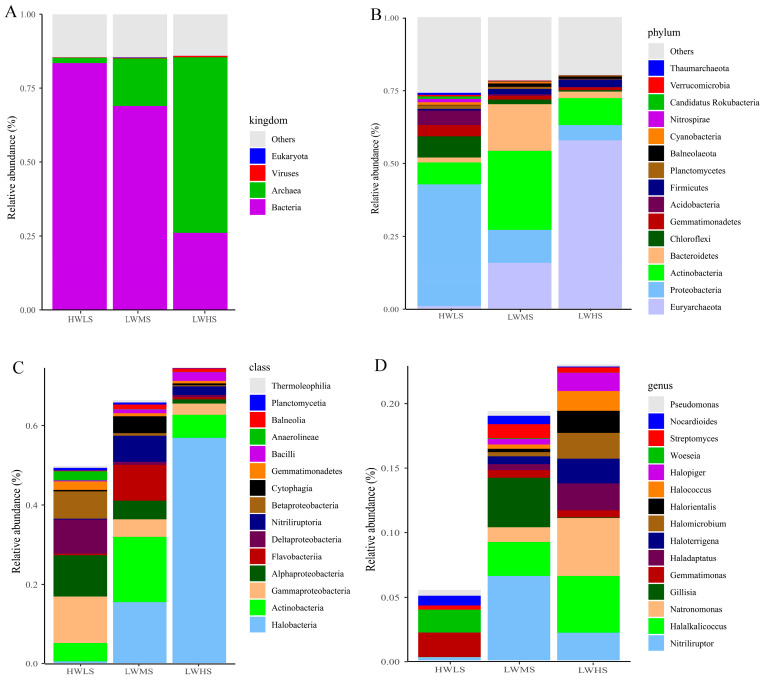
Microbial community composition and relative abundance of dominant taxa in each salt gradient at kingdom (**A**), phylum (**B**), class (**C**), and genus (**D**) level.

**Figure 6:**
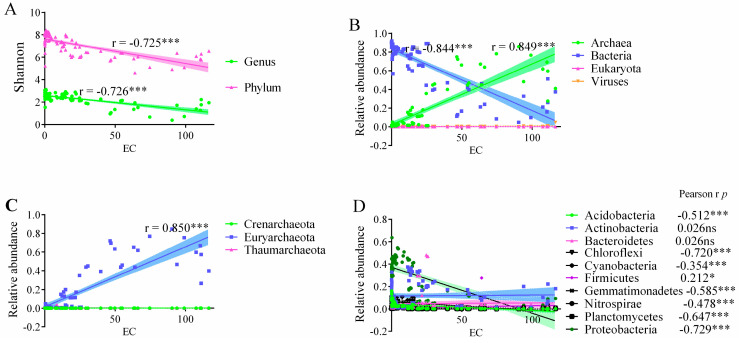
Linear regression between diversity and relative abundance of taxonomic population with salt concentration. (**A**), lineal regression of shannon diversity with EC at genus and phylum level. (**B**), lineal regression of relative abundance of Archaea, Bacteria, Eukaryota, and Virus with EC. (**C**), lineal regression of relative abundance of three phyla in Archaea with EC. (**D**), lineal regression of relative abundance of bacterial phyla with EC. * and *** represent significance at 0.05 and 0.001 level.

The microbial community was dominated by bacteria and archaea across all the samples. The bacteria were most abundant in HWLS soils (83.4%), while their relative abundance decreased in the high-salinized environment, accounting for 69% in LWMS, and 26.0% in LWHS soils. In contrast, the richness of archaea increased with salinity, with relative abundance of 1.88%, 16.07%, and 59.36% in HWLS, LWMS, and LWHS soils (Figure 5 and Figure 6). 

At the phylum and class level, most of the bacterial phyla decreased with salinity, including Proteobacteria (Alpha-, Beta-, Delta-, and Gamma-Proteobacteria), Acidobacteria, Chloroflexi, Cyanobacteria, Gemmatimonadetes, Nitrospirae, and Planctomycetes, whereas relative abundance of Euryarchaeota (class Halobacteria increased from 0.5% to 56.8%), Firmicutes (class Bacilli increased from 0.2% to 2.2%) increased with salinity gradients (Figure 5 and Figure 6). Additionally, some classes’ abundance peaked in the medium-salinized environment (LWMS), such as Actinobacteria (27.1%) and Bacteroidetes (16.05%) (Figure 5).

For the abundant genera, *Gemmatimonas* and *Woeseia* were negatively related to salinity (*p* < 0.05). This resulted in a decrease in the *Gemmatimonas* abundance from 1.74% in HWLS to 0.73 and 0.83 in LWMS and LWHS soils, respectively; while *Woeseia* abundance decreased from 1.74% in HWLS to 0.73 and 0.83 in LWMS and LWHS soils, respectively. Relative abundance of *Nocardioides*, *Streptomyces*, *Salinimicrobium*, *Sphingomonas* and *Bradyrhizobium* were positively related to salt content (pearson r = 0.228, 0.370, 0.338, 0.658, and 0.362, respectively, all *p* < 0.05). *Gillisia* (1.36%) and *Salegentibacter* (0.38%) were also enriched in the LWHS habitat (Figure 5).

A large part of genera exhibited a non-significant trend with salinity, their relative abundance increased from low-salinity to medium-saline soils, then decreased in high-saline soils, such as *Nitriliruptor* (3.57% in LWMS communities), *Halalkalicoccus* (1.51%), *Natronomonas* (2.09%), *Haladaptatus* (0.86%), *Halomicrobium* (0.5%), *Halorientalis* (0.66%), *Halococcus* (0.63%), *Halorubrum* (0.57%), *Halogranum* (0.44%). Moreover, *Pseudomonas*, *Halopiger*, *Desulfuromonas*, and *Haloarcula* were reduced in LWHS soils compared to LWMS and HWLS soils (Figure 5).

**Figure 7:**
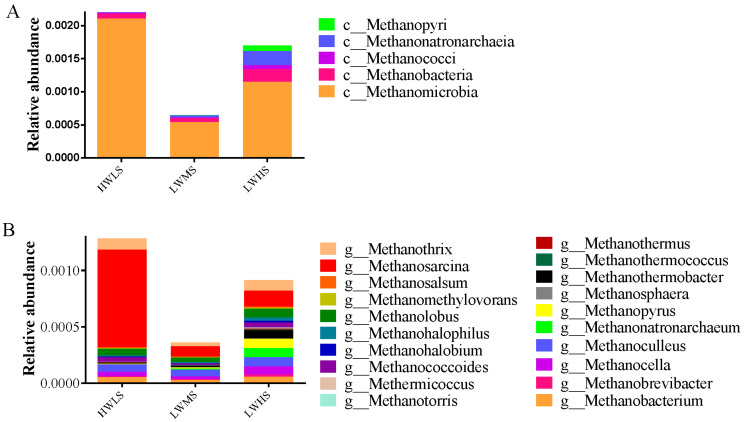
Relative abundance of methanogenic archaea in soil microbial communities at three salinity gradients. (**A**) Class level composition; (**B**) genus level composition.

Five classes of methanogenic archaea were detected, including Methanobacteria, Methanococci, Methanomicrobia, Methanonatronarchaeia, and Methanopyri. Methanobacteria was the most abundant class with an average abundance of 0.16%, its abundance was highest in HWLS habitat, followed by LWHS, and lowest in LWMS habitat. Methanobacteria, Methanonatronarchaeia, and Methanopyri were substantially enriched in LWHS soils. *Methanosarcina* (0.037%), *Methanothrix* (0.008%), *Methanolobus* (0.006%), *Methanobacterium* (0.0045%) were the most abundant methanogenesis genera, and they showed a similar trend to that observed in class Methanobacteria (Figure 7). 

### 3.5. Biomarkers Identification in Each Salinity Group

**Figure 8:**
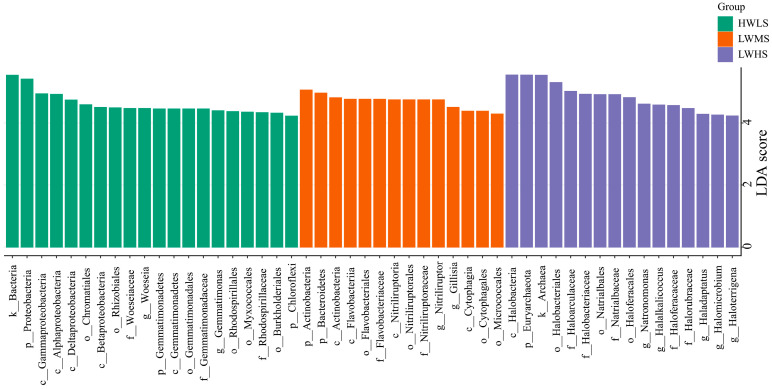
Differences in relative abundance of specific microbial taxa. Taxa changing in relative abundance as determined by linear discriminant analysis (LDA, *p* < 0.05) between the three salinity gradients. Only the top 50 taxonomic groups were shown.

We investigated changes in the relative abundance of specific microbial taxa among the three salt gradients. Our findings revealed a higher number of taxa that differed significantly in relative abundance among the three saline soils, with 20, 14, and 16 taxa being enriched in the HWLS, LWMS, LWHS soil communities, respectively. In the low-saline soils (HWLS), bacteria were enriched, including phyla Proteobacteria (class Alpha-, Beta-, Gamma-, Delta-proteobacteria), Gemmatimonadetes (class Gemmatimonadetes), and Chloroflexi. The abundance of family Woeseiaceae (Woeseia), Gemmatimonadaceae (Gemmatimonas), and Rhodospirillaceae were significantly higher in HWLS. In medium-saline soils (LWMS), phyla Actinobacteria (class Actinobacteriia, Nitriliruptoria) and Bacteroidetes (class Flavobacteria, Cytophagia) were the most enriched taxa. In the high-saline soils, archaea, especially phylum Euryarchaeota, were the most enriched group, with the class Halobacteria being particularly noteworthy (Figure 8).

### 3.6. Co-Occurrence Network Relationships of Soil Microbes along Salinity Gradients

**Figure 9:**
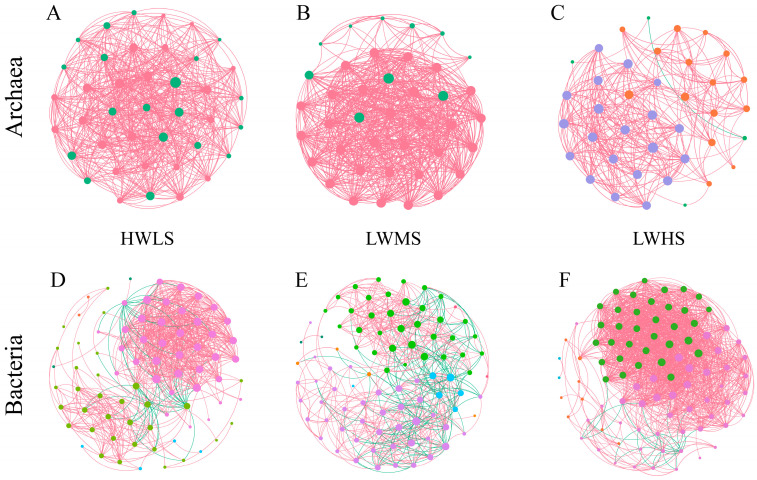
Co-occurrence networks of archaeal and bacterial communities in different salinity groups. Edges represent strong (Spearman |R| > 0.7) and significant (*p* < 0.01) correlations. Colorful nodes represent different modules. (**A**), archaeal co-occurrence network in HWLS community. (**B**), archaeal co-occurrence network in LWMS community. (**C**), archaeal co-occurrence network in LWHS community. (**D**), bacterial co-occurrence network in HWLS community. (**E**), bacterial co-occurrence network in LWMS community. (**F**), bacterial co-occurrence network in LWHS community.

There were significant differences in the co-occurrence network in archaeal and bacterial communities at different salinity gradients. In HWLS soils, archaeal nodes and edges were 40 and 535, respectively, while bacterial nodes and edges were 78 and 636. In LWMS soils, the number of archaeal nodes and edges were 40 and 593, respectively, while the number of bacterial nodes and edges were 90 and 574. In LWHS soil, the number of archaeal nodes and edges were 39 and 284, respectively, while the number of bacterial nodes and edges were 89 and 1412 (Figure 9, Table 1). In the archaeal networks, all of the edges were positive links, and the nodes belonged to c__Halobacteria. In the bacterial network, the positive links accounted for 92%, 72%, and 98% in HWLS, LWMS, and LWHS, respectively. About 81–82% of nodes belonged to Actinobacteria (c__Actinobacteria), Proteobacteria (mainly c__alpha- and gamma- proteobacteria), and Bacteroidetes (mainly c__Flavobacteriia and c__Cytophagia) in each salinity group. The degree of the archaeal network followed normal distributions, whereas the degree of the bacteria network followed bimodal distributions. Modularity values were lower than 0.4 (except for the bacterial network in LWMS), indicating that the constructed networks were absent of modular structures (Table 1). LWMS soil had the greatest number of archaea nodes and edges, making it the most complex network. For bacteria, LWMS soil had the most nodes but the fewest edges, while there were more than twice as many edges in LWHS than in HWLS and LWMS, contributing to the most complex community network in the LWHS.

Key species differed in three salinity gradients. In HWLS soil, the key species in the archaeal network were Candidatus *Halobonum*, *Halosimplex*, *Halomicrobium*, and *Haloparvum*. In LWMS soil, the key archaeal taxa were composed of *Halobiforma*, *Natronobacterium*, *Halovivax*, and *Natronomonas*. While in LWHS soil, the key archaeal species were *Natronomonas*, *Haloparvum*, Candidatus *Halobonum*, *Halosimplex*, and *Natronobacterium*. The key species controlling the bacterial network were *Variovorax*, *Marinobacter*, *Thioalkalivibrio*, *Conexibacter*, *Woeseia*, *Tistlia*, and *Marinimicrobium* in HWLS soil. In LWMS soil, the key bacterial taxa controlling the network were *Halofilum*, *Anaeromyxobacter*, *Thiohalomonas*, *Nocardioides*, *Nocardiopsis*, *Marmoricola*, *Ilumatobacter*, *Salinimicrobium*, *Bacillus*, and *Flavobacterium*. In LWHS soil, the key bacterial taxa were *Mycobacterium*, *Bacillus*, *Altererythrobacter*, *Azospirillum*, *Jiangella*, *Bradyrhizobium*, *Desulfuromonas*, *Gemmatirosa*, and *Gemmatimonas*.

### 3.7. The Relationships between Soil Variables and Community Structure

**Figure 10:**
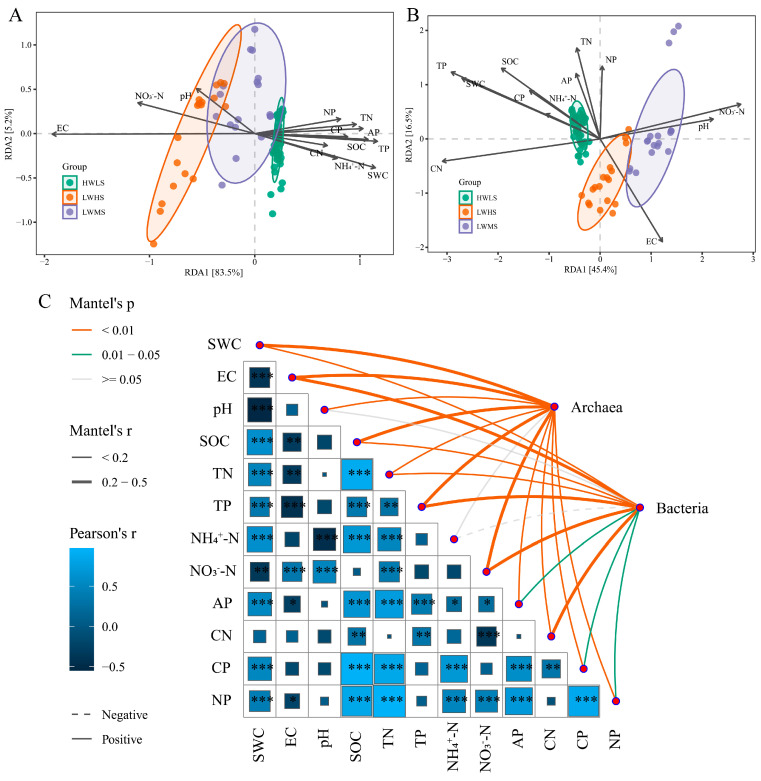
Redundancy Analysis (RDA) and mantel analysis of soil microbial communities with soil variables under different salinity gradients. (**A**) RDA analysis between soil variables and archaeal community; (**B**) RDA analysis between soil variables and bacterial community; (**C**) correlations between soil variables by Pearson analysis and mantel analysis between soil variables and bacterial and archaeal community. *, **, and *** represent significance at 0.05, 0.01, and 0.001 level.

RDA showed that the first two axes totally explained 88.7% of variance of archaeal community composition. pH, EC, NO_3_^−^-N and AP were the most important influential factors (Figure 10A). While 61.9% of total variance was explained by the first two axes of the bacterial community, pH, SOC, NO_3_^−^-N and TP were primary variables structuring bacterial communities. SOC, TN, TP, and AP were negatively correlated with salinity (*p* < 0.05). SWC was negatively correlated with pH and ${NO}_{3}^{-}$-N, but positively correlated with SOC, TN, TP, AP, ${NH}_{4}^{+}$-N, C:P, and N:P (*p* < 0.05) (Figure 10B).

Mantel analysis showed that soil variables had significant effects on soil microbial community composition. All of the soil properties (except ${NH}_{4}^{+}$-N) had a significant influence on the archaeal community. Among these, SOC, SWC, EC, TP, ${NO}_{3}^{-}$-N were the most predominant driving factors (*p* < 0.01); SOC, SWC, EC, TP, TN, ${NO}_{3}^{-}$-N, and AP significantly affected the bacteria community’s structure (*p* < 0.05) (Figure 10C). In summary, SOC, SWC, EC, TP, TN, ${NO}_{3}^{-}$-N, and AP were the primary factors in shaping microbial communities in saline soils.

**Figure 11:**
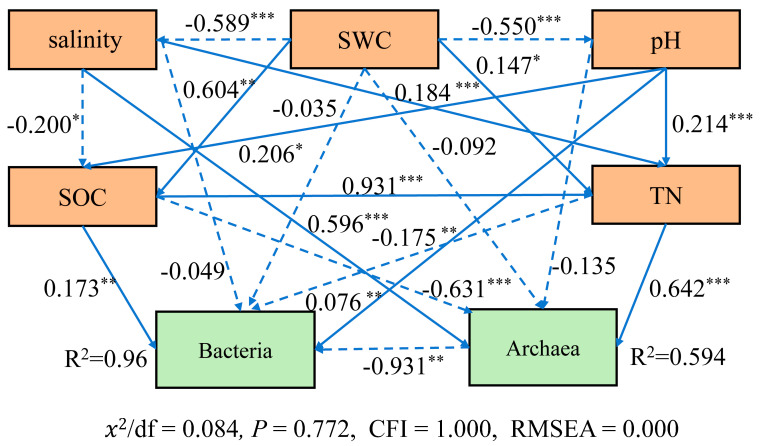
The impacts of soil variables on bacteria and archaea community structures revealed by SEM. *, **, and *** represent significance at 0.05, 0.01, and 0.001 level.

We further selected salinity (EC), SWC, pH, SOC, and TN as the most contributive factors from RDA and mantel analysis to construct a structural equation model. This model was employed to explore the contribution of soil environmental factors to the microbial community under different salinity gradients. SEM revealed that SWC negatively affected salinity and pH (pathway coefficient = −0.589, and −0.550, *p* < 0.001); salinity had a direct negative impact on SOC (coefficient = −0.2, *p* < 0.05); salinity had a direct positive impact on archaea community structure (coefficient = 0.596, *p* < 0.001) but had negative impacts on the bacterial community’s structure indirectly through SOC. Archaea had a direct negative influence on the bacterial community (coefficient = −0.931, *p* < 0.01) (Figure 11). 

## 4. Discussion

### 4.1. The Effects of Salinity on Soil Chemical Properties 

Soil salinization can significantly affect soil microbial activity, nutrient cycling, and soil health [46,47]. This study confirms that the increase in salinity significantly reduces soil organic carbon (SOC), total phosphorus (TP), total nitrogen (TN), and ammonium nitrogen content (${NH}_{4}^{+}$-N), which implies that progression of soil salinization can significantly alter soil fertility and carbon mineralization [48]. It is worth noting that nitrate nitrogen (${NO}_{3}^{-}$-N) increases with salinity, consistent with previous studies [32]. Initially, soil salinization significantly inhibits soil nitrogen mineralization and nitrification rates, but in the later stages of salinization, it significantly enhances soil nitrogen mineralization and nitrification rates [46]. Apart from salinity, soil moisture is also an important factor affecting soil quality and biochemical process. Moderate soil moisture helps promote vegetation growth, sustain organic matter content and nutrient cycling. It can be observed that soil water content (SWC) positively affects SOC, TN, TP, and available phosphorus (AP) content but negatively affects pH. Therefore, the distinct climate causes differences in soil nutrient quantity. The extreme arid climate and high salt concentration in desert regions inhibit soil biochemical process and are unfavorable for soil quality improvement.

### 4.2. The Microbial Community’s Composition at Different Salinity Gradients

Soil salinization affects not only soil nutrients but also the microbiomes inhabiting there. Across a wide range of salinity from coastal to inland China (0.06 to 116 mS/cm), it is confirmed that soil salinization suppresses microbial diversity [19,20]. This is probably due to filtering effects which favor salt-tolerant or halophilic microorganisms but harm salt-sensitive taxa [20,49]. This subsequently causes shifts in the microbial community’s structure [10,16,31]. Our results indicate that the microbial community shows a significant difference at three salt gradients and the notable shifts are that the relative abundance of bacteria decreased and archaea increased. This suggests that a large proportion of bacteria (such as proteobacteria) are replaced by archaea (such as Euryarchaeota) with progression of soil salinization.

In the coastal area where the soil has high water content and low salt concentration (HWLS), the dominant taxa are Proteobacteria, Actinobacteria, Chloroflexi, Gemmatimonadetes, Acidobacteria. These findings are largely consistent with the findings from coastal estuarine wetlands [14] and northeast China [20], except for differences in relative abundance. Proteobacteria abundance dramatically decreased in arid saline soils (more than 70%). In particularly, beta- and delta-proteobacteria are nearly depleted in arid saline soil, as previously reported [20]. Salinity also dramatically decreases the abundance of alpha- and gamma-proteobacteria; only about 40% of them are retained in arid saline soils with low soil water content (LWMS), and 10–25% of them are kept in soil with low water content and high salt concentration (LWHS). These data suggest that the majority members of Proteobacteria are sensitive to salt and drought, while some members of alpha and gamma-Proteobacteria, such as *Pseudomonas* and *Woeseia*, can resist high salt stress and survive in high-salinity soils [20,50]. These bacteria might adopt the organic-solutes-in strategy by increasing the synthesis or absorption of a variety of compatible organic osmolytes or by controlling the flux of ions across cell membranes to resist salt stress [16]. Compared to coastal land, Gemmatimonadetes decreased in arid saline soils, but its abundance remains relatively stable in moderate or hypersaline environments. Gemmatimonadetes has been found to be present in high-salinity–sodicity [20] and a variety of arid soils [50,51]. Chloroflexi can thrive in diverse conditions, from anaerobic to aerobic environments. Some taxa are also capable of fermentation, anaerobic respiration, and the degradation of complex organic compounds. The abundance decline of Chloroflexi may be related to high salt stress and low SOC availability.

The microbial community’s structure in arid saline soils is distinct from that the coastal wetlands. In the LWMS soils, the relative abundance of Proteobacteria decreased, whereas Bacteroidetes, Actinobacteria, Euryarchaeota increased, being the dominant phyla. Class Actinobacteria are widely distributed in soil, being especially predominant in desert habitat, and are contributing to global carbon cycling and plant productivity via soil organic matter decomposition and synthetization of bioactive compounds [17,52]. Actinobacteria is one of the most abundant bacterial phylum, representing its high adaptation capacity to moderate salinity environments [20].

The high salinity and low water levels may introduce ion toxicity and environmental pressures for inhibition of microbial enzyme activity and growth, thus imposing strong selection on microbial community composition [53]. Under stronger salt stress in LWHS, Proteobacteria, Bacteroidetes, and Actinobacteria are further suppressed, in contrast, the archaea abundance increases. This results in Euryarchaeota becoming the most abundant phylum dominated by class Halobacteria, similar to the findings in QTP salt lakes [11]. Halobacteria are extremophiles that can thrive in high-salt environments such as salt flats, salt lakes, and salt mines. This is due to their preference for environments with high salinity, often requiring high salt concentrations to grow optimally, and they possess specialized adaptations to survive in high-salt environments [54,55]. They maintain osmotic balance through the accumulation of compatible solutes or by adjusting the intracellular ion concentrations to match the external salinity. Their unique adaptations make them well-suited for survival in harsh environments [54]. Some species are capable of anaerobic respiration using alternative electron acceptors, such as nitrate or sulfur compounds [56]. Even though high salinity suppresses a wide range of bacterial taxa, some bacterial members in classes Gammaproteobacteria, Actinobacteria, Bacilli, Planctomycetia, such as *Pseudomonas*, *Woeseia*, *Nocardioides*, *Gemmatimonas*, may have developed strategies to adapt to high-salinity environments.

Methanogenic archaea are responsible for methane (CH_4_) production through processes including acetoclastic methanogenesis, hydrogenotrophic methanogenesis, and methylotrophic methanogenesis. Methanogenic microorganisms are found in wetlands, sediments, springs and hydrothermal vents [57,58], as CH_4_ is mainly produced in natural environments such as wetlands, oceans, and sediments [59]. Furthermore, methanogenic archaea were identified in arid land, including classes Methanobacteria, Methanopyri, Methanococci and Methanomicrobia. However, the total proportion of methanogenic archaea and the dominant class Methanomicrobia were significantly lower in arid land than that in coastal wetlands. However, the other four classes of Methanobacteria, Methanopyri, Methanococci, and Methanonatronarchaeia were significantly higher in arid land that those in coastal wetlands (*p* < 0.05). Increasing salinity inhibits hydrogenotrophic methanogens but enhances acetoclastic methanogenesis in Tibetan lake sediments [60]. Consistently, we found that the acetoclastic methanogenesis genus Methanosarcina is significantly correlated with salinity (Pearson r = −0.209, *p* < 0.05), no significant correlations were detected between the relative abundance of Methanosarcina and SOC, pH, and SWC. However, the response of methylotrophic methanogenesis archaea to salinity is different. Higher salinity decreased the abundance of Methanosarcinales, but increased the abundance of Methanosphaera. In general, the different methanogen responds to salt in different pattern, and methanogenesis archaea decreased in arid land, which implies a lower CH_4_ emission potential in arid saline soils than that in coastal wetlands. 

### 4.3. The Effects of Salinity on Microbial Community Network

Complex and multifaceted interactions are presenting within soil microbial communities, including both positive (e.g., mutualistic) and negative (e.g., competitive, antagonistic) interactions, which can influence the microbial communities’ structure, function, and their responses to environmental changes [61,62]. Salinity gradients can influence the structure of microbial co-occurrence networks by altering the relationships among different microbial taxa. As observed above, the microbial community structure, the network complexity and key species of bacteria and archaea changed with salinity. With salinity increases, the bacteria community’s network complexity decreases, as evidenced by less links, lower average degree, and average clustering coefficients from HWLS to LWMS. In environments with low salinity (HWLS), bacterial communities may display complex co-occurrence patterns with many positive and negative associations, reflecting a diverse array of interactions including mutualism, competition, and commensalism. As salinity increases, these complex networks may be disrupted, and new interactions may emerge. The positive link ratio decreases from 92% in HWLS to 72% in LWMS, then increases to 98% in LWHS. Additionally, the network complexity in LWHS was enhanced, indicating that the interactions established by the remaining halotolerant or halophilic bacteria were enhanced to develop a more synergistic relationships in the bacterial community, which might play an important role in resistance to salinity stress, improving the capacity of microbial communities for material cycling, energy flow, and information transfer [63]. Zheng et al. reported that salinity enhanced the interactions among bacteria in high-salt environments [8]. In contrast, the archaea community network showed an opposite pattern compared to bacteria. The complexity peaked in the LWMS. In addition, almost all the links were positive, indicating that soil archaeal communities prefer to coexist in a synergistic mutualistic manner with strength of salt stress. These confirm that the soil archaea and bacteria communities have different responses to soil salinity.

Keystone species have a critical impact on the ecosystem that is disproportionate to their abundance. We find that there are different key species on different salt gradients. The key archaeal taxa in three salinity gradients are *Halosimplex*, *Halomicrobium*, *Haloparvum*, *Halobiforma*, *Natronobacterium*, *Halovivax*, and *Natronomonas*. The key bacterial species in three salinity gradients are different. *Variovorax*, *Marinobacter*, *Thioalkalivibrio*, *Conexibacter*, *Woeseia*, *Tistlia*, and *Marinimicrobium* are key species in HWLS. In LWMS soil, the key taxa include *Halofilum*, *Anaeromyxobacter*, *Thiohalomonas*, *Nocardioides*, *Nocardiopsis*, *Marmoricola*, *Ilumatobacter*, *Salinimicrobium*, *Bacillus*, and *Flavobacterium*. In LWHS soil, the key species are composed of *Mycobacterium*, *Bacillus*, *Altererythrobacter*, *Azospirillum*, *Jiangella*, *Bradyrhizobium*, *Desulfuromonas*, *Gemmatirosa*, and *Gemmatimonas*. Many of these key species play important roles in the function and stability of the ecosystem. *Bacillus* have diverse metabolic capabilities and are involved in processes such as nutrient cycling, organic matter decomposition, phosphate solubilization, nitrogen fixation, and plant growth promotion [64,65]. *Azospirillum* are known for their ability to nitrogen fixation [66] which helps improve soil fertility and reduce the need for synthetic nitrogen fertilizers. *Nocardioides*, *Nocardiopsis* and *Marmoricola* in Nocardioidaceae are commonly found in various environments such as soil, water, and plant rhizospheres [67,68,69,70] which play important roles in organic matter decomposition, nitrogen cycling, and antibiotic production [71]. *Jiangella* are capable of producing ß-glucosidase, ß-xylosidase, and a-larabinofuranosidase, which have potential in lignocellulose-based biorefining [72]. Other bacteria, such as *Gemmatimonas* [73], *Bradyrhizobium* [74,75,76], *Anaeromyxobacter* [77], *Thiohalomonas* [78], and halophilic archaeon including *Halococcus* [79], *Halorubrum* [80], *Halobiforma* [81], and *Halomicrobium* [82] play key roles in biochemical cycle of organic matter, nitrogen cycling, sulfur-oxidizing.

### 4.4. The Impacts of Soil Variables on Microbial Community

Salinization is the primary environmental determinant affecting the composition of microbial communities [10,16,31]. Apart from salt, other edaphic variables also influence microbial communities directly or indirectly, and this differs among ecosystem type, climate and soil conditions. For example, the salinity, pH, and nutrients can optimize the microbial community structure in desert ecosystems [83]; the dissolved oxygen, pH, total nitrogen, and ${PO}_{4}^{3-}$ are primary driving factors in plateau salt lakes [11]; soil organic matter is the primary influencing factor of microbial communities in coastal saline-alkaline areas [5].

This study shows that soil EC, SWC, and pH significantly influence soil TN and TP. Salt directly affects archaeal community, but indirectly affects bacteria community through SOC. A previous study reported that salinity causes shifts in abundance and community structure of nitrate reducers (denitrification and dissimilatory nitrate reduction to ammonium) and the effect of salinity on these organisms may be mediated by alterations in soil organic matter availability [84]. Soil moisture and salinity together exert an environmental pressure on composition and functioning of microbial community [85,86]. Higher salinity would also cause the water availability to be limited for soil microorganisms [87]. This may explain the significant relationships between bacterial community and soil moisture (mantel test *p* < 0.05). Substantial research has demonstrated that soil pH is a critical driver for the microbial community’s diversity and structure [88]. However, there are also studies reporting a weak impact of pH on the bacterial community compared with soil salinity [14]. We found significant correlations between pH and the bacterial community, the archaeal community, SOC, and TN, suggesting a relatively strong impact of soil pH on the soil microbial community. We also observed that pH affects archaeal community indirectly through TN, but directly affects bacterial community. In general, EC, SWC, pH, TN and SOC are the main driving factors for the composition of soil archaea and bacteria communities. TN and SOC directly influence microbial community structure. EC and SWC affect bacterial or archaeal community directly or indirectly through TN or SOC.

### 4.5. Clues for Reclamation of the Degraded Saline Soils

Soil salinity acts as a negative driver influencing the microorganisms composition and biogeochemical processes, leading to changes in nutrient cycling processes such as nitrogen fixation, mineralization, and organic matter decomposition [14] which might have cascading effects on soil fertility and plant health. Our research has found that soil salinity levels affect SOC and TN; SOC directly affect sthe soil bacterial community, which indirectly affects the archaeal community through TN. This also reflects that enhancing soil fertility can improve soil microbial community metabolism and thus improve saline-alkali soils [89]. It is important to note that the approaches for improving saline-alkali soils by enhancing fertility may differ between the low-salinization and high-salinization soils due to different environmental factors in the ecological zones [9,90]. Improvement in soil fertility by adding external nutrients and addressing issues such as poor permeability and aeration in saline-alkali soils may be effective in the eastern coastal region but not for the arid Xinjiang region with a higher salt concentration and low water moisture. Increasing evidence shows that inoculating salt-tolerant microorganisms provides an effective way to improve saline-alkali soils and plant growth [28,32,91,92]. The key taxa identified in each salinity gradient, and some highly abundant taxa, such as *Woeseia*, *Pseudomonas*, *Nitriliruptor*, *Desulfuromonas* have abilities in organic compound decomposition, nitrogen and sulfur cycling, and bioactive compound production [93]. These beneficial microorganisms are potential biofertilizers that have important applications in biotechnology, degraded soil reclamation, and agriculture in arid saline regions.

This study provides insights into the taxonomic and community-level changes to salinity, but some limitations exist which need to be addressed in the future, such as clarifying the functional potential or gene expression patterns that could provide further insights into the mechanistic responses of the microbiomes to salinity; investigating the seasonal or temporal variations in response to salinity changes; incorporating targeted experiments (e.g., microcosm studies, manipulation of salinity levels) that could help validate and strengthen the observed relationships between soil variables and microbial community’s dynamics.

## 5. Conclusions

In this study, we investigate the effects of salinity on the microbial community’s structure on three salinity gradients from two climatic regions with distinct precipitation and salinity conditions. Our research confirms that soil salinity has a negative effect on soil quality, as all nutrient contents except nitrate nitrogen decrease significantly with increasing salinity. Salinity has a great effect on the soil microbial community’s structure, leading to the shifts from bacteria-dominant communities to archaea-dominant communities. In addition, salinity changed the interactions within the microbial community; the network complexity and keystone taxa altered with salinity. LWMS soil had the most archaea nodes and edges, making it the most complex network. LWHS had the most complex bacterial community network. EC, SWC, pH, TN, and SOC are the main driving factors for the composition of soil archaeal and bacterial communities. TN and SOC directly influence microbial community structure. EC, SWC, and pH affect bacterial or archaeal community directly or indirectly through TN or SOC. Salt directly affects the archaeal community, but indirectly affects the bacterial community through SOC. pH affects the archaeal community indirectly through TN, but directly affects the bacterial community. The high abundance and key taxa observed in the saline soils have potential for reclamation of the degraded saline soils, as many of them are involved in organic compounds’ metabolisms and cycles. Therefore, this study not only deepens our insights regarding the effects of salinity on the microbial community’s composition, but also provides potential clues and resources for microbial-based remediation of saline soils.

## Figures and Tables

**Table 1:** Topological characteristics parameters of microbial co-occurrence networks.

	Archaea			Bacteria		
	HWLS	LWMS	LWHS	HWLS	LWMS	LWHS
Nodes	40	40	39	78	90	89
Edges	535	593	284	636	574	1412
Positive edges percent	100	100	100	92	72	98
Average degree	26.75	29.65	14.56	16.31	12.76	31.73
Clustering coefficient	0.76	0.95	0.82	0.79	0.55	0.81
Average path length	0.98	1.09	1.56	1.99	1.91	1.65
Network diameter	2	2	4	5	5	5
Density	0.69	0.76	0.38	0.21	0.14	0.36
Modularity	0.12	0.08	0.36	0.33	0.41	0.20

## Data Availability

The raw sequence data of arid land soil samples have been deposited to the NCBI Sequence Read Archive (SRA) under project PRJNA876575. The newly generated raw sequence data of coastal soil samples have been deposited to the National Genomics Data Center under project PRJCA017624.

## Supplementary Materials

The following supporting information can be downloaded at: https://www.mdpi.com/article/10.3390/microorganisms12071268/s1, Table S1. The geographic information of sampling location used in present study; Table S2. The statistics of metagenomic sequencing and assembly of microbial community in each soil sample; Figure S1. PCoA based on the Bray–Curtis distance matrices indicating β-diversity differences between three salinity groups at phylum level.
