# Supplementary figures and images for: Shifts in Microbial Community Structure and Co-occurrence Network along a Wide Soil Salinity Gradient
Source: Microorganisms. 2024 Jun 22;12(7):1268. doi: 10.3390/microorganisms12071268 (PMC11278679; doi:10.3390/microorganisms12071268)

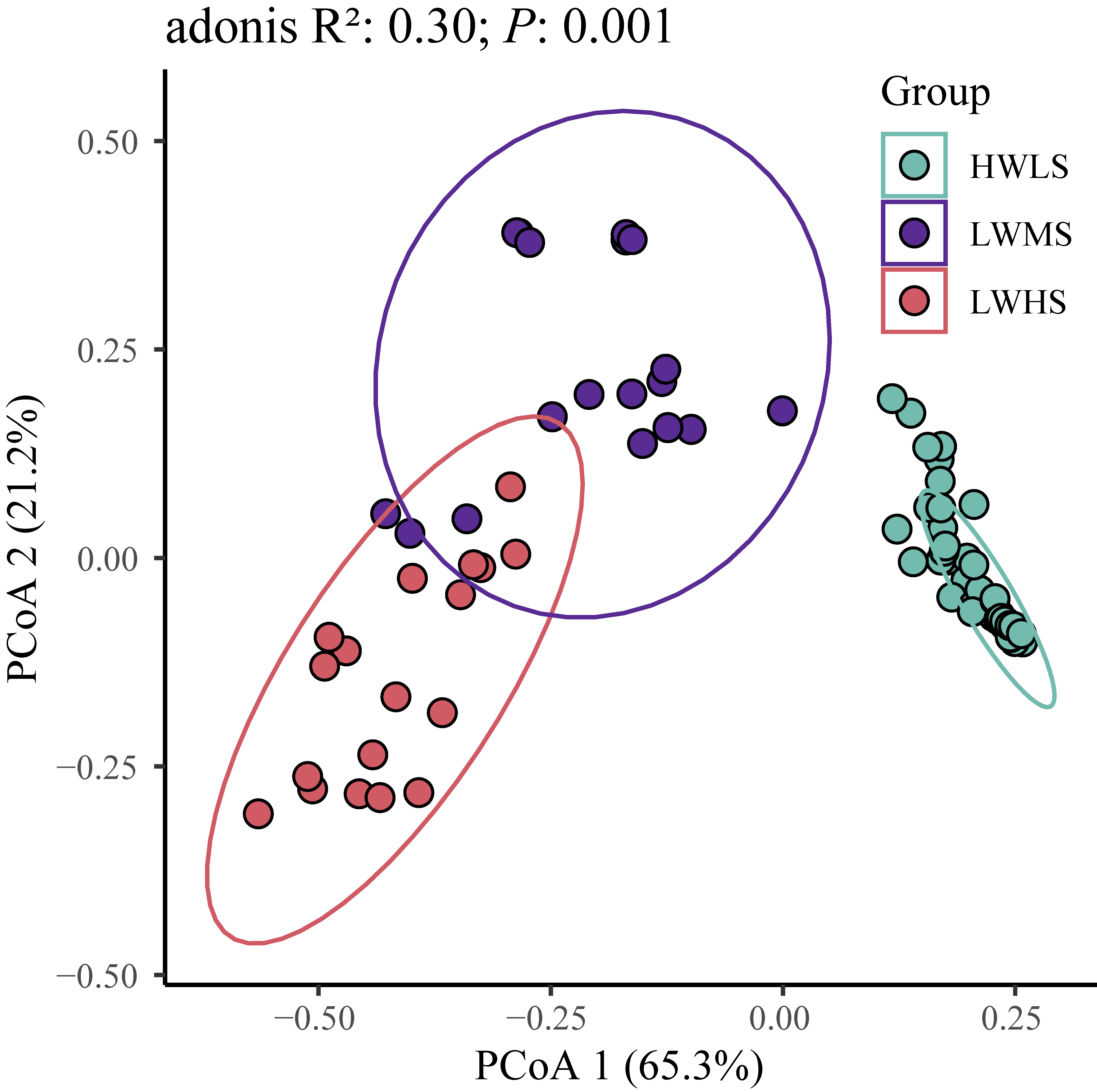

Supplement: Supplementary file 1 [file microorganisms-12-01268-s001.zip › Figure S1.tiff]
